# Green Economy Development Progress in the Republic of Buryatia (Russia)

**DOI:** 10.3390/ijerph19137928

**Published:** 2022-06-28

**Authors:** Alexey Bilgaev, Erzhena Sadykova, Anna Mikheeva, Taisiya Bardakhanova, Svetlana Ayusheeva, Fujia Li, Suocheng Dong

**Affiliations:** 1 Institute of Geographic Sciences and Natural Resources Research, Chinese Academy of Sciences, Beijing 100101, China; bilgaev@igsnrr.ac.cn; 2 Baikal Institute of Nature Management, Siberian Branch of the Russian Academy of Sciences, 670047 Ulan-Ude, Russia; ersadykova@binm.ru (E.S.); asmiheeva@binm.ru (A.M.); tbard@binm.ru (T.B.); asvetl@binm.ru (S.A.); 3 Innovation Academy for Green Manufacture, Chinese Academy of Sciences, Beijing 100190, China

**Keywords:** Republic of Buryatia (Russia), Lake Baikal, green economy, composite index, forecast

## Abstract

Under current conditions, the green economy concept has received a comprehensive response in achieving the sustainable development of regions. However, measuring green economic development progress is dynamic, quantitatively characterized by indicators reflecting various aspects. The difficulty lies in a comprehensive environmental sustainability assessment in a context that includes the territory’s environmental, social, and economic factors. The study aimed to assess the progress of the Republic of Buryatia’s (Russia) “green” economic development. The proposed methodology for constructing a composite index is based on five dimensions’ aggregation—resource efficiency, environmental efficiency, environmental quality of life, natural assets, and institutional factors. The composite index helped generalize the complex processes of the region’s environmental–socio–economic development. Its main feature is the reflection of the environmental specificity of the territory. We built a mid-term forecast of the composite and sub-indices, determined their future trend, and assessed the opportunities and conditions for the fastest transition of the Republic of Buryatia to a green economy. The developed composite index is a key tool for regulating green economic development progress, determining prospects, and region management. This paper attempts to fill the gap in a comprehensive assessment of the Republic of Buryatia’s current situation using a composite index.

## 1. Introduction

Conventional economic development (pollution, environmental degradation, and biodiversity loss) used to be viewed as unavoidable consequences of economic growth [1,2]. The slowdown of economic growth has consequences on the well-being of the population. In achieving sustainability—improving the standard of living, rational use, and reproduction of natural resources—environmental protection is becoming essential for a region’s development. During the last decades, various ideas and concepts have emerged from academia, industry, or political movements to support sustainability transformations by attempting to reconcile economic, social, and environmental goals [3]. One such concept is the green economy. The “Green economy” term was first coined during the late 1980s [4] by Pearce et al. [5] in the “Blueprint for a Green Economy” report [6] and mainstreamed after the 2012 United Nations Conference on Sustainable Development in Rio de Janeiro (Rio + 20) [7]. The international literature contains many definitions relating to the green economy [8]. The United Nations Environment Program (UNEP) defines the green economy as ‘improved human well-being and social equity, while significantly reducing environmental risks and ecological scarcities’; ‘low carbon, resource-efficient, and socially inclusive’ [9]. In the green economy, growth in income and employment should be driven by public and private investments that reduce carbon emissions and pollution, enhance energy and resource efficiency, and prevent the loss of biodiversity and ecosystem services that result in improved human well-being and social equity, while significantly reducing environmental risks and ecological scarcities [10]. The green economy concept is well established in the political sphere, and it appears in many policy agendas of international institutions and, currently, is more related to concepts linked to weak sustainability (i.e., energy efficiency or pollution control) [11]. As the green economy is continually being made and remade, its shape and contours are contingent upon and open to transformation [12], and each region chooses its strategy for achieving a green economy that promotes economic growth, considering climate change problems (green economy and green jobs will be key for economic recovery while simultaneously battling climate change and driving job growth) [13,14]. Green economy progress is a multi-dimensional process that measures several indicators representing its different dimensions [15]. The assessment should be based on composite indices because particular indicators describing certain phenomena do not show the comprehensive idea of the research object. Composite indices are the aggregation of all component indicators describing a multi-dimensional and often complex issue [16]; they combine multi-dimensional processes into simplified concepts that are often used for advocacy and policy consumption [17]. Such indices have been used in many contexts from economics to engineering, energy efficiency evaluation [18], planning and innovations [19], and environmental sciences [20] and might also help set policy directions [21].

There are many studies on the comprehensive assessment of green economic development using indicator assessment methods [22,23,24] in the world practice and an increasing number of studies on the greening of the economy of the Republic of Buryatia [25,26,27]. However, the problem of finding sustainable ways to preserve unique natural objects, including Lake Baikal, has not yet been sufficiently studied. Most research focuses on theoretical approaches to the region’s transition to a green economy and empirical analysis of the environmental situation [28,29,30]. The problems of quantifying the progress of the Republic of Buryatia’s transition to a green economy and measuring the policy effectiveness have not been studied. Therefore, it is necessary to carry out a comprehensive assessment of its environmental–socio–economic development, which would allow measuring concrete green economic progress.

We aimed to assess the Republic of Buryatia’s current progress in the transition to a green economy and its development prospects. Accordingly, we developed original and novel methodology for diagnosing and assessing progress in the development of the green economy in the Republic of Buryatia by the calculation of a composite index using the adopted set of individual indicators based on the OECD methodological approach [17] considering the environmental, socio–economic, and institutional factors affecting it. The composite index is a key toolkit that allows for determining current trends in the region and the pace of transitioning to a “green economy”.

We formed five dimensions with primary data (resource efficiency, environmental efficiency, environmental quality of life, natural assets, and institutional factors) and then normalized and weighted the data to calculate the sub-indices of each dimension. The main feature of the proposed composite index is the reflection of the environmental specificity of the territory. We built a mid-term forecast of the composite and sub-indices, determined their future trend, and assessed the opportunities and conditions for the fastest transition of the Republic of Buryatia to a green economy. The environmental–socio–economic system of Buryatia possesses a specific potential and uses the available opportunities for dynamic development. We chose the neural network model as a forecasting method [31,32], as it better solves problems for which statistical methods perform poorly. The results made it possible to generalize the complex processes of the region’s environmental–socio–economic development, obtain quantitative assessments of the region’s economic state from the green economic development perspective, and determine the further prospects and regional management directions. The paper makes recommendations for policy changes and proposed regulatory mechanisms to facilitate the transition to a green economy. We attempted to fill the existing gap in a comprehensive assessment of the current situation in the Republic of Buryatia and the green economy progress. The theoretical contribution of the study is that the green economy can effectively perform its functions within the specified parameters and can have high adaptive properties in response to ongoing environmental changes. Therefore, the greening region’s economy should become a mandatory object of management and regulation. The practical significance of the study results lies in the fact that the developed original methodology for assessing the green economy progress of the Republic of Buryatia using a composite index will provide solid support in making government management decisions in the greening of the economy. Therefore, the obtained practical assessments and directions in regulating the development of the region’s green economy have a nationwide, strategic nature that meets the needs of optimal management of the environmental–socio–economic development of the Republic of Buryatia. The study can be helpful for local authorities in similar regions with unique natural systems, such as Lake Baikal.

## 2. Materials and Methods

### 2.1. Study Area

The Republic of Buryatia (capital city is Ulan-Ude) is a federal subject of the Russian Federation with an area of 351 thousand km^2^ (2.04% of the territory of Russia) and a population of 984.6 thousand people (0.67% of the total population of Russia). Buryatia is in the center of Asia (south of Eastern Siberia), with territory stretching from the southwest to the northeast, determining the different economic management conditions. Mountain ranges are extensive and deeply characterize the relief and almost closed inter-mountain basins. The sharply continental climate of Buryatia formed under the dry and cold climate of the northern regions, the hot and dry Mongolian deserts, and the humid Pacific Ocean. The winter is cold with dry frosts, and the major snowfalls occur in November and December. The spring is windy with frost and occasion precipitation. The summer is short, with hot days and cool nights and heavy rains in July and August; autumn comes without a sharp change in weather. The feature of the climate of Buryatia is the long duration of sunshine, i.e., 19–22 h, which is no less than in the southern regions of Russia. The runoff capacity of Buryatia’s rivers is 98 km^3^; 94.3 thousand m^3^/year per capita (almost three times higher than the average for Russia); and 279.8 thousand m^3^/year per 1 km^2^ of territory. In total, the basin of Lake Baikal accounts for 61% of the river runoff of the Republic [27]. Currently, there is no clear answer as to how sustainable green development correlates with the realities of the socio–economic development of Russian regions with various branches of economic specialization and environmental conservation requirements. This problem is especially acute for the Republic of Buryatia, which occupies an exceptional place in Russia and the world due to Lake Baikal and its unique natural features. The proposed methodological approach was tested in the Republic of Buryatia as a model area for green economic development in the Russian Federation.

### 2.2. Research Methodology

**Figure 1:**
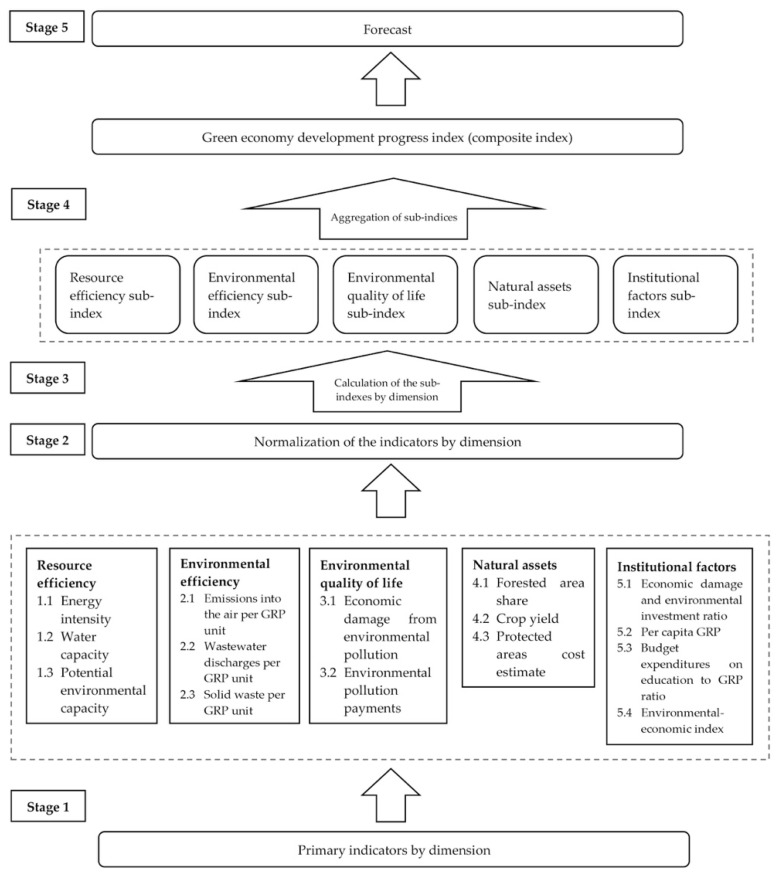
Stages of the developed methodology.

Following the proposed recommendations for selecting primary indicators of the composite index [33] and creating sub-indices by dimension, we developed the following method (Figure 1):

(1) Selection of indicators to calculate the composite index—data sets;
(2) Clarification of the relative importance of individual indicators that determine the values of sub-indices;
(3) Data normalization;
(4) Weighting;
(5) Calculation of sub-indices;
(6) Aggregation of sub-indices into a composite index;
(7) Building a composite index forecast;
(8) Visualization of results

This study formed the following five dimensions of the Republic of Buryatia’s “green” development system. The ***Resource efficiency*** is represented by the energy intensity, water capacity, and potential environmental capacity—generalized characteristics of the territory, quantitatively corresponding to the maximum technogenic load that the totality of recipients and ecosystems can withstand for a long time without violating their structure and functionality [34,35]. The ***Environmental efficiency*** includes the GRP unit’s emissions, wastewater discharges, and solid waste. These two indicator groups reflect the need to carefully use the environment and natural resources and cover production aspects that economic models and accounting systems rarely quantify. We propose the use of the economic damage from the environmental pollution indicator, which includes additional costs (for treatment and medical care, income decrease) due to environmental deterioration [36], to characterize the ***environmental quality of life***. The ***Environmental pollution payments*** indicate compensation for the environmental damage caused by organizations and individuals engaged in any natural resource use-related activity. The ***Natural assets*** base is necessary to achieve ecological balance. It includes the forested area share, crop yield, and protected areas’ cost estimate (determined by the under-received volume of GRP, since these areas are completely or partially withdrawn from the economic turnover). The ***Institutional factors*** include activities and policy instruments that affect the environment’s quality and sustainable green development formation. Therefore, we proposed the indicators that make it possible to assess the policy efficiency: economic damage and environmental investment ratio (indicate the efficiency of the existing environmental management economic mechanism); per capita GRP; budget expenditures on education to GRP ratio; and the environmental–economic index. The inclusion in this indicator group, the environmental–economic index, is relevant for resource-oriented regions, including the Republic of Buryatia. It allows assessing the impact of the extractive industry volume on the GRP, environmental pollution, and ecosystem degradation level, determining the regions’ environmental development trajectory and identifying the adjusted net savings structure [37].

Continuing a comprehensive study [33], we attempted to build a composite index based on indicators (Table 1). The main difference between the proposed set of indicators and the original is its specific purpose and application in assessing the region’s progress toward green development and determining the prospects for the environmental–socio–economic development of the Republic of Buryatia. Initial data for the Republic of Buryatia were obtained from environmental and socio–economic situation state reports, the Russian Federation, regions of Russia, and the Republic of Buryatia statistical yearbooks [38,39].

### 2.3. Composite Index Calculation Methodology

We determined the calculated and statistical indicators and combined them into five dimensions. We normalized the data using the min–max method, which is especially useful for obtaining unified values from 0 to 1. To implement these transformations, we determined the *X_min_* and *X_max_* values for each sub-index *X*. We proposed the use of an empirical approach, i.e., for *X_min_* and *X_max_* values, the min and max values were taken, respectively, among the values of this variable observed across time in Russian regions.

The normalized value for each indicator included in the sub-index was calculated as shown below.

The calculation for the positive correlation (larger the value of *X*, the higher the quality) was as follows:

(1) $$X_{i}=\frac{x_{i}-x_{min}}{x_{max}-x_{min}}$$

The calculation for the negative correlation (the larger the value of *X*, the lower the quality) was as follows:

(2) $$X_{i}=\frac{x_{max}-x_{i}}{x_{\;max}-x_{min}}$$

where *X_i_*—normalized indicator value; *i*—index of indicators (*i* = 1, … n); *x_i_*—*i*th indicator’s value; *x_max_* and *x_min_*—maximum and minimum values of *x_i_*.

After the indicators are normalized, the next step is weighting and aggregation. In the study, the weighting procedure was carried out by assigning equal weights for all indicators within each dimension. This approach assumes that all variables used to build the composite index are equally significant. However, it can also hide the absence of a statistical or empirical basis for choosing weights, i.e., when there is not enough knowledge about the relationships between components, or experts fail to reach a consensus. This approach does not mean abandoning the weighing procedure but assumes that all weights are the same. Each dimension’s weight is 1/5. For example, the first dimension is resource efficiency, it has three indicators, and their weights are distributed equally—each indicator weight is (1/5)/3 = 1/15 (Table 2).

In the next stage, we calculated dimension sub-indices, and our choice assumed that the indicators are interchangeable (higher values of other indicators can compensate for the low value of one indicator).

Sub-index *I_i_* in year *t* was calculated as follows:

(3) $$I_{it}=\sum X_{it}*\;f_{it}$$

Five sub-indices by dimension were aggregated into a single composite index as the sum of the values of these indices as follows:

(4) $$I_{composite\;t\;}=\sum I_{it}^{fi}\;$$

where $I_{t\;}$—composite index of year *t* (index value is between 0 and 1); *I_it_*—sub-index *i* in year *t*; *f_i_*—weight of *i*, which must have a positive value; the sum of all weights equals 1.

### 2.4. Neural Network Method

The problem of forecasting the development of the Republic of Buryatia as a model territory for green economic development in the Russian Federation is a priority. The forecasting results are necessary to substantiate the goals and objectives of the further development of the economy of the model area and the development and justification of program activities in the transition to a “green economy” and sustainable development.

We chose artificial neural networks with automatic topology construction, particularly the forecasting method proposed in [31,32] and previously described in [25]. The forecasting process using a neural network consisted of the following steps: preparing the data, training a neural network using a genetic algorithm, and forecasting the composite index and its sub-indices. The neural model made it possible to consider the Republic of Buryatia’s unique features due to Lake Baikal’s presence and to build a more realistic forecast. This study tested this method on the example of the Republic of Buryatia and showed promising results.

## 3. Results

The Republic of Buryatia is one of the Russian industrial–agricultural regions. The area of the Republic of Buryatia is 351 thousand km^2^, 2.04% of the Russian Federation. There is a positive dynamic of population growth—in 2019, the population was 984.6 thousand, 0.67% of the Russian Federation population. However, the Republic of Buryatia and the entire east of Russia is characterized by a low population density—2.8 people per km^2^. In Buryatia, the urban population grew over the study period. Thus, in 2019, the proportion between the urban and rural populations was 59.2/40.8, while in 2010, the ratio was 58.5/41.5.

Table 3 shows indicators characterizing the economic and environmental situation dynamics in the study model area.

In 2019, the GRP of the Republic of Buryatia amounted to 4415.3 million dollars (0.3% of the total Russian GRP). There has been a consistent increase in GRP in recent years, and in 2019 the increase was 7.8% compared to the previous year. GRP per capita in 2019 increased by the same 7.8% compared to 2018, while the indicators of the republic were more than two times lower than the total for Russia. Over the study period, the indicators of the emissions, wastewater discharges, and waste volume changed non-uniformly. In recent years, emissions increased by 6.4%, while there has been a decrease in the wastewater discharges and waste volumes by 11.8 and 9.8%, respectively, which is more likely due to structural changes in the region’s economy. In 2019, the volume of investments in environmental protection decreased by more than half (52.7%) and amounted to 3.5 million dollars—0.1% of the total Russian investment in environmental protection, compared to 2010 (84.1%).

### 3.1. Composite Index

The use of the composite index to analyze and assess progress toward the transition to green economic development made it possible to generalize the region’s complex environmental and socio–economic development processes. The main feature of the proposed composite index is the reflection of the environmental specifics of the territory. It includes 15 indicators grouped into five dimensions (Table 1). After defining the list of indicators, we built the dimension’s sub-indices by normalization. The purpose of normalization is to bring all values of variables to the same scale of their measurement. We used a min–max normalization method, which is especially useful for obtaining unified values from 0 to 1. Then, we weighed indicators by giving equal weights to them within each dimension (Table 2). Thus, we obtained the values of normalized indicators (Table 4). Finally, we carried out a dynamic analysis of indicators over the 10 years to assess the positive or negative trends along the green economic development path.

Table 5 shows the calculated sub-indices and the composite index for the analyzed period.

In 2019, compared to 2010, the growth rate of the resource efficiency sub-index was 102.4%. Environmental efficiency indicators showed a negative picture, and the rate of decline in the sub-index for the analyzed period was 50.7%. A relatively favorable situation developed in the social sphere of the region, as evidenced by the observed positive trends in the change in the values of the environmental quality of the life sub-index—the overall growth rate was 115.9%. The increase in the natural assets’ sub-index showed that maintaining the natural balance was still in a state of conservation. As a result of the analysis of the sub-indices by dimensions, it can be noted that institutional factors had the most significant impact on the value of the composite index—the growth rate of the institutional factors’ sub-index was 297.2%.

**Figure 2:**
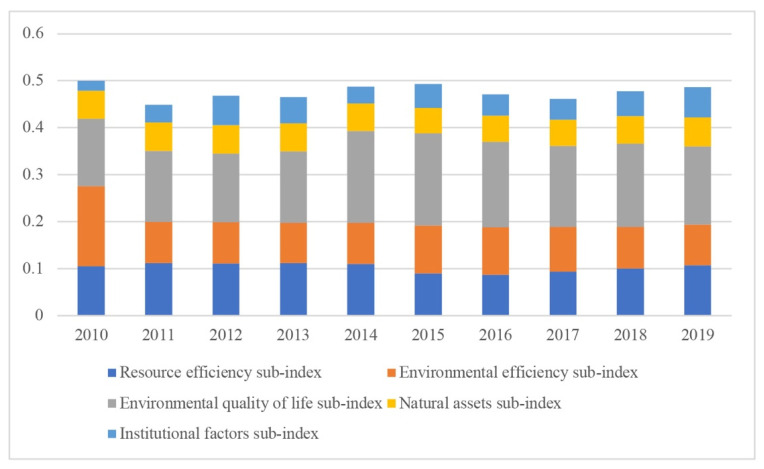
Composite index structure, 2010–2019.

The analysis showed a general decrease in the composite index by 2.8%. In this case, it is necessary to consider the structural shifts during the analyzed period. Figure 2 shows the dynamics of the structural shifts in the composite index.

We assessed the structural shift intensity by tempo indicators. We observed the most dramatic structural shifts in the environmental sphere. The share increased from 34.5% in 2010 to 52.2% in 2019 due to a significant increase in waste. In addition, there was a sharp increase in the institutional sphere share in the composite index structure, from 4.36% to 13.31%. There was also an increase in the environmental quality of life share by 19.2%.

### 3.2. Composite Index Forecast

Forecasting the green economic development of the region is the prediction of the future environmental–socio–economic state of the regional system, an integral part of state regulation of the regional economy, which determines the direction of development of the region and its structural components. The forecasting is necessary to substantiate the goals and objectives of transitioning to green economic development and rationalizing using limited natural resources. State program activities and priorities in the region’s future development are specified based on a reasonable forecast. We conducted experiments on the medium-term forecast of the resulting composite index and its sub-indices. Table 6 shows the medium-term (2019–2026) forecast of the composite index for the Republic of Buryatia, developed based on the adopted system of indicators.

**Figure 3:**
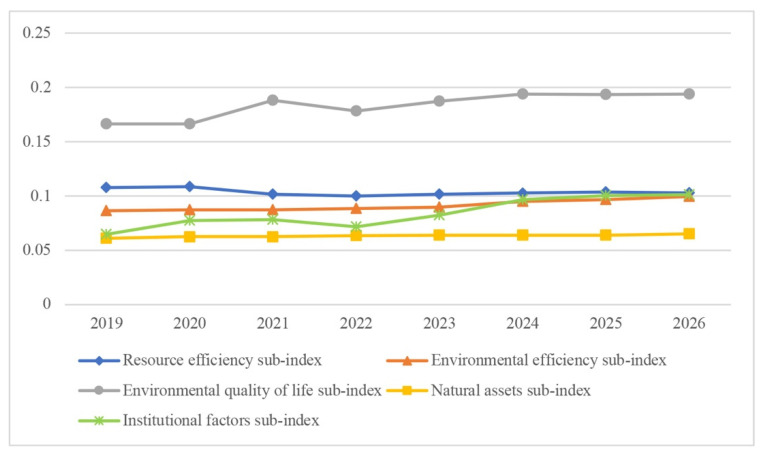
Sub-indices’ forecast.

The resource efficiency index will decrease by 4.7% during the forecast period. On the other hand, the highest growth rate is expected in environmental policy—a growth rate of 156%, followed by social equity—116.5%, environmental efficiency—115.5%, and the natural assets’ sub-index—6.3%—compared to 2019 (Figure 3).

**Figure 4:**
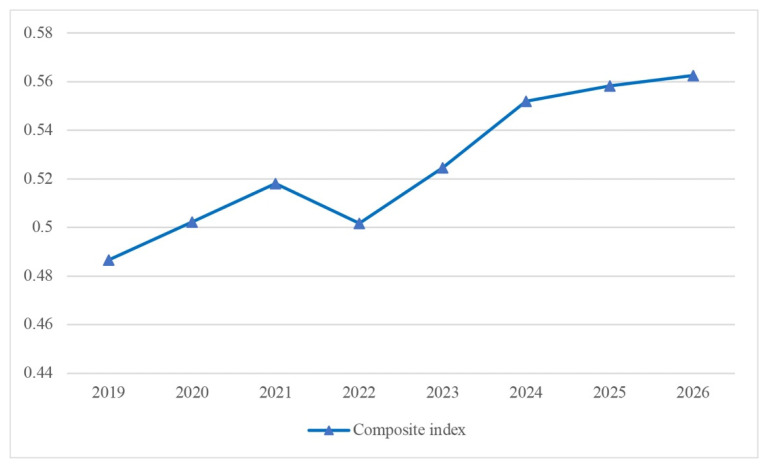
Composite index forecast.

In general, the composite index value will increase by 15.6% by 2026 compared to 2019 (Figure 4).

## 4. Discussion

1. The analysis of the indicators characterizing the environmental and economic situation in the Republic of Buryatia showed that, despite the positive dynamics of GRP indicators in recent years, the growth of GRP itself is not evidence of a favorable economic situation in the Republic, where the well-being of the region depends on the extraction and sale of minerals. This problem characterizes the Russian economy, where the mining sector is the main sector. Buryatia’s economic basis is the manufacturing industry—mechanical engineering and metalworking, mining (gold, coal, uranium), building materials, timber, electrical equipment production, and food and light industries. It inevitably leads to various kinds of waste and, consequently, to environmental problems in the region.

Energy decarbonizations and emissions result from the economic activity of households and enterprises, cheap, low-quality coal, and an increase in the number of vehicles. The primary pollutants are concentrated in the industrial centers of the Republic—Ulan-Ude, Gusinoozersk, and Selenginsk. The “Baikal factor”, which imposes restrictions and special requirements on economic activity in general, including the electric power industry, determines the conduct of environmental activities in this industry. Shifting from fossil fuels to zero-carbon sources has become one of the priorities [40]. “We must end fossil fuel pollution and accelerate the renewable energy transition before we incinerate our only home,” U.N. Secretary-General Antonio Guterres said. “Time is running out.” [41]. The Republic of Buryatia has excellent potential for developing renewable energy (strong winds and a high number of sunny days). The experience of China, the world leader in renewable energy sources, can be helpful. China already built 71.67 GW of new wind farms in 2020 [42] and constructed the first ultra-high voltage electricity line to transmit only carbon-free electricity, boosting renewable energy consumption while reducing idle capacity (10,000 jobs) [43]. The Federated States of Micronesia developed grid-connected wind and solar power plants—300 kilowatts (kW) of roof-mounted solar photovoltaics (PV) and 1.4 megawatts (MW) of wind turbines [44]. In 2022, seven solar power plants will operate in the Republic of Buryatia in 6 out of 23 districts, with a total capacity of 145 MW [45]. Clear and robust policies, transparent processes, public support, and the availability of modern energy transmission systems are key to accelerating the uptake of wind and solar energy technologies [46].

The largest polluters of water bodies are enterprises that produce and distribute electricity, gas, and water. Therefore, it is necessary to introduce modern technologies to reduce the anthropogenic pressure on water resources [47]—construction of treatment facilities using innovative solutions. This requires coordination and consideration of the interests of all subjects of water use, the adoption of preventive measures, and the improvement of the economic mechanism of water use. Waste formation in Buryatia is observed in precious and rare metal mining, coal extraction, and cement, lime, and gypsum production. Therefore, it is necessary to create a database on waste and processing methods, introduce a monitoring system, and use economic incentives for waste to solve the waste disposal problem [25].

It is important to note that increasing levels of greenhouse gases in the atmosphere due to human activities are a major driver of climate change [48]. Without immediate and deep emissions reductions across all sectors and regions, it will be impossible to keep warming below 1.5 °C [49]. Protecting and restoring ecosystems and managing land sustainably can reduce annual net greenhouse gas emissions by more than 7 gigatons by 2030 [50].

Investments are one of the most important factors determining economic development and significantly impacting environmental situations. During the study period, there were sharp changes in growth rates and structural shifts in investments aimed at protecting the environment. On the other hand, organizations and enterprises are not interested in financing environmental projects that require investment, as in the long run, this will decrease the efficiency indicators and, in general, their competitiveness. The main reason for the unsatisfactory state of water bodies and the atmosphere is insufficient funding for environmental protection measures. However, the funds allocated by the budget, by the enterprises themselves, are far from enough to reverse the negative trends that have developed in the environmental protection system, especially when preserving a unique water body—Lake Baikal. Greater investments are needed to ensure a just transition—including in people’s skills training, research and innovation, and incentives to build supply chains through sustainable practices that protect ecosystems and cultures [46]. In this regard, it is necessary to implement measures aimed at activating investment and innovation processes. The region’s future and the fate of Baikal depend on success in solving this problem.

2. An assessment of the current progress in the green economic development of Buryatia by the composite index construction revealed the development trends of the republic. The composite index showed that during the study period of 2010–2019, there was a positive trend in indicators characterizing resource efficiency—the index growth was 2.4%. Indicators of natural productivity demonstrate this as an inverse indicator of natural intensity, where there is a tendency to increase the efficiency of using natural resources per unit of output. The main factor that had the most significant negative impact on the state of the natural environment in the republic was the increase in the production and consumption of waste. In the Republic of Buryatia, waste disposal is one of the main environmental problems. Mining generates a large amount of waste. The index of institutional factors had the greatest impact on the composite index values. This indicates that the existing economic mechanism of environmental activities has a positive effect. The results of the composite index calculation showed that the studied model area—the Republic of Buryatia—provides economic growth due to high amounts of natural assets. On the other hand, a low level of resource efficiency and lack of funding for environmental protection measures lead to an increase in financial losses associated with environmental pollution and necessitate the improvement of institutional relations in terms of developing economic incentives to reduce economic damage from environmental pollution.
3. The results of the composite index forecast showed consistent growth, which will reach 15% by 2026. In general, in the Republic of Buryatia, by 2026, all sub-indices show a positive dynamic, except for the resource efficiency sub-index (the rate of decline was 95.3% compared to 2019), due to the growth of the republic’s economy, so the dynamics here are unstable. It is a temporary compromise based on the reasonableness of the combination of economics and environmental interests. The growth of the environmental efficiency sub-index (15.5%) shows that, according to the accepted scale, there is a trend toward a green economy transition, that is, environmental restrictions imposed on the production and economic activities of industrial, agricultural, and other enterprises of the Republic of Buryatia have had a positive impact on the environmental situation. Thus, achieving the appropriate values for each sub-index by dimension should become a priority guideline in regulating the sustainable development of the Republic of Buryatia. Of course, a necessary condition for implementing this forecast is the country’s positive direction of political and economic stability. The development of the Republic of Buryatia is possible by using the competitive advantages of the model territory of green sustainable development (the presence of Lake Baikal, the border position, the cultural center of Buddhism, and the center of ecotourism). Considering that Lake Baikal is an “environmental strategic resource” whose significance goes beyond the national framework, Russia and the world community should be objectively interested in the accelerated green development of the Republic of Buryatia.
4. The main disadvantages of the Russian environmental policy are the lack of environmental priorities in the economic strategy (lack of a clear strategy in the environmental field, weak environmental management, gaps in the legislative framework, low level of funding for environmental protection measures, poor innovative activity in the environmental field, and low level of attention to the environmental culture of the population [51]. The positive factors influencing the preservation of the purity of Baikal water include the following. Lake Baikal has a special status, fixed at the federal and world levels as a UNESCO World Natural Heritage Site (1996). Therefore, the protection of Lake Baikal is not only a local but also a national and global problem. Adopted law on “Protection of Lake Baikal” (federal law of the Russian Federation, 1 May 1999, N 94-FZ on “Protection of Lake Baikal”) allocated three zones with different protection regimes and levels of anthropogenic pressures on the Baikal natural territory—central, buffer, and zone of atmospheric influence. Enterprises located within ecological zones conduct a regulated natural assessment of the economic activity, observing environmental standards and requirements.

The Republic of Buryatia accounts for 73% of the Baikal basin. The environmental regulation regime has been in force for more than 50 years, affecting the socio–economic development of the region [52]. The Republic of Buryatia has undergone reforms that have radically changed its socio–economic structure and the environmental and social global processes—climate change, globalization, and optimization of socio–economic processes. At the same time, the Republic’s specific conditions, i.e., low level of economic development (according to the results of 2021, the Republic of Buryatia is ranked 72 out of 85 in terms of the socio–economic situation of Russian regions [53]); special environmental requirements for all types of life activities that limit economic activity in Buryatia; low population density; and border location, significantly complicate the transition to sustainable green development. Thus, for the Republic of Buryatia, the measures taken by state regulatory bodies are essential since the business does not receive sufficient support for an independent transition to a green economy and is at the stage of forming a response to the transition. At the moment, Buryatia is in a situation where the state must stimulate business. The Republic’s budget is 50 percent of the expense of the federal budget, and government plays the leading role in choosing the direction of the Republic’s development. Thus, the “Protection of Lake Baikal” law has a double effect; on the one hand, restrictions make it possible to maintain the purity of Baikal water, and on the other hand, restrictions deprive the enterprise of the opportunity to develop, thereby creating social tension, and unemployment is growing.

Therefore, we propose the implementation of an integration model project for the transition from a raw material orientation to an innovative path for effective interaction in the development of a macroregion based on a “green” economy, primarily through the transition to clean energy in the territories adjacent to Lake Baikal—(Baikal Natural Territory). Policies and incentives must align with current local conditions for renewable energy deployment, both in terms of technical potential and how effectively the current power market compensates for new clean energy generation, identifying the suitable geographies and areas for renewable energy development [54]. It is necessary to involve financial systems through tax and other incentives, including banks and other public and private financial institutions. Moreover, it is essential to ensure commitment to accelerating the transition to green economic development and accountability. Therefore, we propose the creation of a special inter-regional Fund for the Green Development of the Baikal Region and the launch of the state program “Green Development of the Baikal Region”. Regional authorities will perform the regulatory function within the Baikal region [27]. First, this should determine the forms and methods of state support; determine the list of organizations eligible for subsidies; and establish the procedure for granting subsidies and reporting. The main idea is to restructure incoming public funds; the point is to redistribute financial flows from polluting “brown” industries to “green” ones [10]. “Renewables are the only path to real energy security, stable power prices, and sustainable employment opportunities”.—Antonio Guterres, UN Secretary-General. Shifting subsidies from fossil fuels to renewable energy cuts emissions and contributes to sustainable economic growth, job creation, better public health, and more equality, particularly for the poor and most vulnerable communities around the world [46]. It is necessary to smooth out the greening of production, considering business interests by creating regulatory legal acts that will minimize the possible decrease in the profitability of enterprises. Refurbishment of existing assets (conventional power plants) helps to adapt to new conditions and meet the evolving needs of the system—increasing energy system flexibility [55]. Therefore, subsidies, credits, and tax breaks must subsidize technical re-equipment to introduce green technologies and enterprises’ industries. Second, it is necessary to provide enterprises with personnel to organize refresher and retraining courses. Third, we need to involve scientific organizations through grants for green developments and the analysis and forecasting of environmental, social, and economic processes; this will help quickly and accurately understand the current situation and develop development strategies for each enterprise.

In general, implementing the proposed strategic directions will make it possible to achieve economic stability on the path of the green sustainable development of the Republic of Buryatia.

## 5. Conclusions

The study results show that the developed methodology for the Republic of Buryatia’s green economic progress assessment by calculating a composite index with a multi-level indicator system allows the study of model territories to determine the region’s current trends and the green economy transition pace. A composite index is a key tool in regulating the region’s green development, reflecting the significant aspects of the environmental–socio–economic situation and its prospects. For the Republic of Buryatia, the achievement of quantitative assessment parameters and the resulting forecast of the composite index will provide information support in solving further development problems and a better understanding of how environmental conservation will affect the economy and population.

The obtained practical assessments and directions in regulating the development of the region’s green economy are of a national, strategic nature, meeting the needs of optimal management of the development of the Republic of Buryatia. Implementing the proposed strategic directions will ensure economic stability on the Republic of Buryatia’s green sustainable development path. This experience can be helpful for local authorities in similar regions with unique natural systems striving to green the economy.

In further studies, it is necessary to improve the proposed approach to studying regional environmental–socio–economic trends, considering the green economy transition pace and the emergence of associated risks and furthermore, to carry out work on the scientific substantiation of the regional economic restructuring to introduce the principles of green development into the activities of economic entities.

## Figures and Tables

**Table 1:** Assessment directions and indicators for calculating the composite index.

Dimension	Indicators	Variable
Resource efficiency X_1_	Energy intensity, kW·h/USD X_11_	Electricity consumption volume/GRP
	Water capacity, m^3^/USD, X_12_	Water consumption volume/GRP
	Potential environmental capacity, thousand TOE X_13_	Extensive parameter determined by the territory size (km^2^) and its volume (km^3^) × Content of the main ecologically significant substances in the environment (t/km^3^, t/km^2^) × Environment volume or mass multiple renewal rate (year)
Environmental efficiency X_2_	Emissions into the air per GRP unit, TOE/USD X_21_	Absolute value of emission of pollutants into the air indicator/GRP
	Wastewater discharges per GRP unit, TOE/USD X_22_	Absolute value of the wastewater discharge indicator/GRP
	Production and consumption waste per GRP unit, TOE/USD X_23_	Absolute value of the production and consumption waste volume indicator/GRP
Environmental quality of life X_3_	Economic damage from environmental pollution, (mln USD) X_31_	Environmental damage by a unit of pollutants’ reduced mass (USD/TOE) × Reduced mass of pollutants (TOE)
	Environmental pollution payments (mln USD) X_32_	Statistical indicator
Natural assets X_4_	Forested area share (%) X_41_	Forested area/Total territory
	Crop yield, (dt/ha) X_42_	Statistical indicator
	Protected areas’ cost estimate, (bln USD) X_43_	GRP/(100 − Protected area share in the total territory) × Protected area share in the total territory
Institutional factors X_5_	Economic damage and environmental investment ratio (times) X_51_	Environmental damage/Environmental investments
	Per capita GRP (USD) X_52_	GRP/Population
	Budget expenditures on education to GRP ratio (%) X_53_	Budget expenditures on education/GRP
	Environmental–economic index (%) X_54_	Adjusted net savings (ANS)/GRP × 100% ANS = Gross fixed capital formation − Investments in fixed assets from mineral extraction − Mineral extraction gross value volume − Environmental damage + Budget expenditures on human capital development + Capital expenditures on environmental protection + Protected areas’ cost estimate

**Table 2:** Distribution of weights by indicators.

	Weight
**Resource efficiency X_1_**	**1/5**
Energy intensity, kW·h/USD X_11_	1/15
Water capacity, m^3^/USD, X_12_	1/15
Potential environmental capacity, thousand TOE X_13_	1/15
**Environmental efficiency X_2_**	**1/5**
Emissions into the air per GRP unit, TOE/USD X_21_	1/15
Wastewater discharges per GRP unit, TOE/USD X_22_	1/15
Production and consumption waste per GRP unit, TOE/USD X_23_	1/15
**Environmental quality of life X_3_**	**1/5**
Economic damage from environmental pollution, (mln USD) X_31_	1/10
Environmental pollution payments (mln USD) X_32_	1/10
**N** **atural assets X_4_**	**1/5**
Forested area share in the total territory, % X_41_	1/15
Crop yield, dt/ha X_42_	1/15
Protected areas’ cost estimate, (bln USD) X_43_	1/15
**Institutional factors X_5_**	**1/5**
Economic damage and environmental investment ratio (times) X_51_	1/20
Per capita GRP (USD) X_52_	1/20
Budget expenditures on education to GRP ratio (%) X_53_	1/20
Environmental–economic index (%) X_54_	1/20

**Table 3:** Characteristics of the Republic of Buryatia.

Years	GRP (mln USD)	Per Capita GRP (USD)	Emissions into the Air (Thousands T)	Wastewater Discharges (Thousands m^3^)	Production and Consumption Waste (Thousands T)	Investments in Environmental Protection (mln USD)
2010	4381.2	4513.9	95.2	42,400	16,727.6	22.3
2015	3242.7	3307.6	109.0	39,200	50,230.7	4.8
2018	4096.7	4163.7	90.6	34,600	80,503.6	7.5
2019	4415.3	4484.4	96.4	30,500	72,593.7	3.5

**Table 4:** Normalized indicators, 2010, 2012, 2014–2019.

Sub-Indices	2010	2012	2014	2015	2016	2017	2018	2019
Energy intensity	0.04865	0.05285	0.05212	0.03948	0.03815	0.04193	0.04521	0.04927
Water capacity	0.05379	0.05590	0.05537	0.04779	0.04661	0.04912	0.05278	0.05571
Potential environmental capacity	0.00291	0.00291	0.00291	0.00291	0.00291	0.00291	0.00291	0.00291
**Resource efficiency sub-index**	0.10535	0.11165	0.11040	0.09018	0.08766	0.09396	0.10089	0.10788
Emissions into the air per GRP unit	0.05255	0.01224	0.01328	0.02184	0.02004	0.01906	0.01437	0.01418
Wastewater discharges per GRP unit	0.05207	0.00987	0.01103	0.01823	0.01881	0.01371	0.01274	0.01042
Production and consumption waste per GRP unit	0.06551	0.06501	0.06350	0.06199	0.06219	0.06288	0.06074	0.06171
**Environmental efficiency**	0.17013	0.08712	0.08781	0.10207	0.10104	0.09564	0.08785	0.08631
Economic damage from environmental pollution	0.09649	0.09544	0.09486	0.09663	0.09730	0.09604	0.09612	0.09648
Environmental pollution payments	0.04713	0.05115	0.09967	0.09981	0.08365	0.07600	0.08100	0.07000
**Environmental quality of life sub-index**	0.14361	0.14659	0.19454	0.19645	0.18094	0.17204	0.17712	0.16648
Forested area share	0.05103	0.05128	0.05133	0.05136	0.05125	0.05145	0.05111	0.05113
Crop yield	0.00723	0.00734	0.00599	0.00158	0.00429	0.00328	0.00712	0.00881
Protected areas’ cost estimate	0.00118	0.00144	0.00141	0.00086	0.00080	0.00103	0.00110	0.00119
**Natural assets** **’** **sub-index**	0.05945	0.06007	0.05873	0.05380	0.05634	0.05576	0.05933	0.06114
Economic damage and environmental investment ratio	0.00968	0.04928	0.02312	0.04167	0.03792	0.03611	0.04470	0.04944
Per capita GRP	0.00381	0.00522	0.00501	0.00198	0.00167	0.00290	0.00328	0.00376
Budget expenditures on education to GRP ratio	0.00033	0.00030	0.00042	0.00021	0.00021	0.00047	0.00052	0.00070
Environmental–economic index	0.00798	0.00807	0.00750	0.00656	0.00542	0.00476	0.00375	0.01088
**Institutional factors** **’** **sub-index**	0.02180	0.06286	0.03606	0.05042	0.04522	0.04424	0.05225	0.06478

**Table 5:** Dynamics of the sub-indices and composite index, 2010–2019.

Year	Resource Efficiency Sub-Index	Environmental Efficiency Sub-Index	Environmental Quality of the Life Sub-Index	Natural Assets’ Sub-Index	Institutional Factors’ Sub-Index	Composite Index
2010	0.10535	0.17013	0.14361	0.05945	0.02180	0.50034
2011	0.11250	0.08753	0.15049	0.06133	0.03753	0.44939
2012	0.11165	0.08712	0.14659	0.06007	0.06286	0.46830
2013	0.11181	0.08581	0.15214	0.05971	0.05604	0.46551
2014	0.11040	0.08781	0.19454	0.05873	0.03606	0.48754
2015	0.09018	0.10207	0.19645	0.05380	0.05042	0.49291
2016	0.08766	0.10104	0.18094	0.05634	0.04522	0.47121
2017	0.09396	0.09564	0.17204	0.05576	0.04424	0.46165
2018	0.10089	0.08785	0.17712	0.05933	0.05225	0.47744
2019	0.10788	0.08631	0.16648	0.06114	0.06478	0.48659

**Table 6:** Composite index forecast.

Year	Resource Efficiency Sub-Index	Environmental Efficiency Sub-Index	Environmental Quality of the Life Sub-Index	Natural Assets’ Sub-Index	Institutional Factors’ Sub-Index	Composite Index
2019	0.10788	0.08631	0.16648	0.06114	0.06478	0.48659
2020	0.10862	0.08738	0.16657	0.06239	0.07724	0.50220
2021	0.10176	0.08738	0.18837	0.06239	0.07814	0.51805
2022	0.10003	0.08832	0.17836	0.06339	0.07158	0.50169
2023	0.10161	0.08963	0.18739	0.06390	0.08214	0.52468
2024	0.10261	0.09495	0.19395	0.06385	0.09664	0.55200
2025	0.10354	0.09648	0.19368	0.06399	0.10051	0.55821
2026	0.10279	0.09972	0.19399	0.06499	0.10107	0.56256

## Data Availability

No new data were created or analyzed in this study. Data sharing is not applicable to this article.
